# Decoding microglia responses to psychosocial stress reveals blood-brain barrier breakdown that may drive stress susceptibility

**DOI:** 10.1038/s41598-018-28737-8

**Published:** 2018-07-26

**Authors:** Michael L. Lehmann, Thaddeus K. Weigel, Hannah A. Cooper, Abdel G. Elkahloun, Stacey L. Kigar, Miles Herkenham

**Affiliations:** 1 Section on Functional Neuroanatomy, Intramural Research Program, National Institute of Mental Health, NIH, Bethesda, MD 20892 USA; 2 Division of Intramural Research Programs Microarray Core Facility, National Institutes of Health, Bethesda, MD 20892 USA

## Abstract

An animal’s ability to cope with or succumb to deleterious effects of chronic psychological stress may be rooted in the brain’s immune responses manifested in microglial activity. Mice subjected to chronic social defeat (CSD) were categorized as susceptible (CSD-S) or resilient (CSD-R) based on behavioral phenotyping, and their microglia were isolated and analyzed by microarray. Microglia transcriptomes from CSD-S mice were enriched for pathways associated with inflammation, phagocytosis, oxidative stress, and extracellular matrix remodeling. Histochemical experiments confirmed the array predictions: CSD-S microglia showed elevated phagocytosis and oxidative stress, and the brains of CSD-S but not CSD-R or non-stressed control mice showed vascular leakage of intravenously injected fluorescent tracers. The results suggest that the inflammatory profile of CSD-S microglia may be precipitated by extracellular matrix degradation, oxidative stress, microbleeds, and entry and phagocytosis of blood-borne substances into brain parenchyma. We hypothesize that these CNS-centric responses contribute to the stress-susceptible behavioral phenotype.

## Introduction

Disease occurrence in individuals can appear to be stochastic. Susceptibility is affected by a combination of genetic and environmental factors as well as internal states. This is notably true for mental disorders, which occur largely unpredictably against a backdrop of genetic predispositions interacting with physiological and psychological perturbations that contribute to disease occurrence or non-occurrence in particular individuals. Human research has shown that individuals have varying vulnerabilities to depression triggered by environmental challenges^1,2^. This separation of outcomes is seen in animal studies in which chronic psychological stress induces anxiety and depressive-like states in some but not all animals, leading to the designation of animals as either susceptible or resilient to the effects of stress^3–8^. Thus, individual differences in stress-regulatory circuits can dramatically affect vulnerability to illness.

There is an emerging literature relating depression and its underpinnings in chronic stress to alterations in the immune system^9–12^. We wondered whether different behavioral outcomes to stress might have a neuroimmunological component. Stress susceptibility might have peripheral^13^ or central immunological origins^14,15^, the latter in microglia, the brain’s immune cells^16^. Microglia have been reported to be activated by acute and chronic psychological stress^17–19^, possibly due to the generation of damage-associated molecular pattern signals (DAMPs) within the brain^9^. Microglia can contribute to the effects of chronic stress on brain function and behavioral responses^17,19,20^ and are thought to be a major source of proinflammatory cytokines that contribute to a generally toxic environment in the brain^14^. In addition, microglia–neuron interactions regulate the maturation, function and modification of synapses, as well as adult neurogenesis^21^. Chronic stress enhances microglial phagocytosis of cellular elements, including axon terminals and dendritic spines^22^. These activities may underlie stress-induced mood declines.

To examine the factors associated with the development of resistance versus susceptibility to the depressive-like effects of chronic social defeat (CSD) stress in mice, we asked whether stress-induced induction of mood disorders might be related to stress-induced central immune responses, i.e., whether there is a correlation between degree of dysregulated affect and microglial immune activity. We examined and compared activation states of microglia from normal mice and from stressed mice segregated into susceptible and resistant phenotypes according to behavioral scores taken during the CSD procedure. Microglia were isolated from whole brains and subjected to gene expression analysis by microarray. Bioinformatic analysis of transcriptional profiles indicated that microglia from susceptible (CSD-S) mice were considerably different than those from resilient (CSD-R) mice, whose profiles were similar to those of home-cage control (HC) mice. Functional assays were performed to validate the microarray findings. The data showed that susceptibility to CSD was associated with increased phagocytosis and oxidative stress in microglia and opening of the blood-brain barrier (BBB) characterized by isolated microbleeds.

## Results

### Behavioral analysis and population stratification

**Figure 1:**
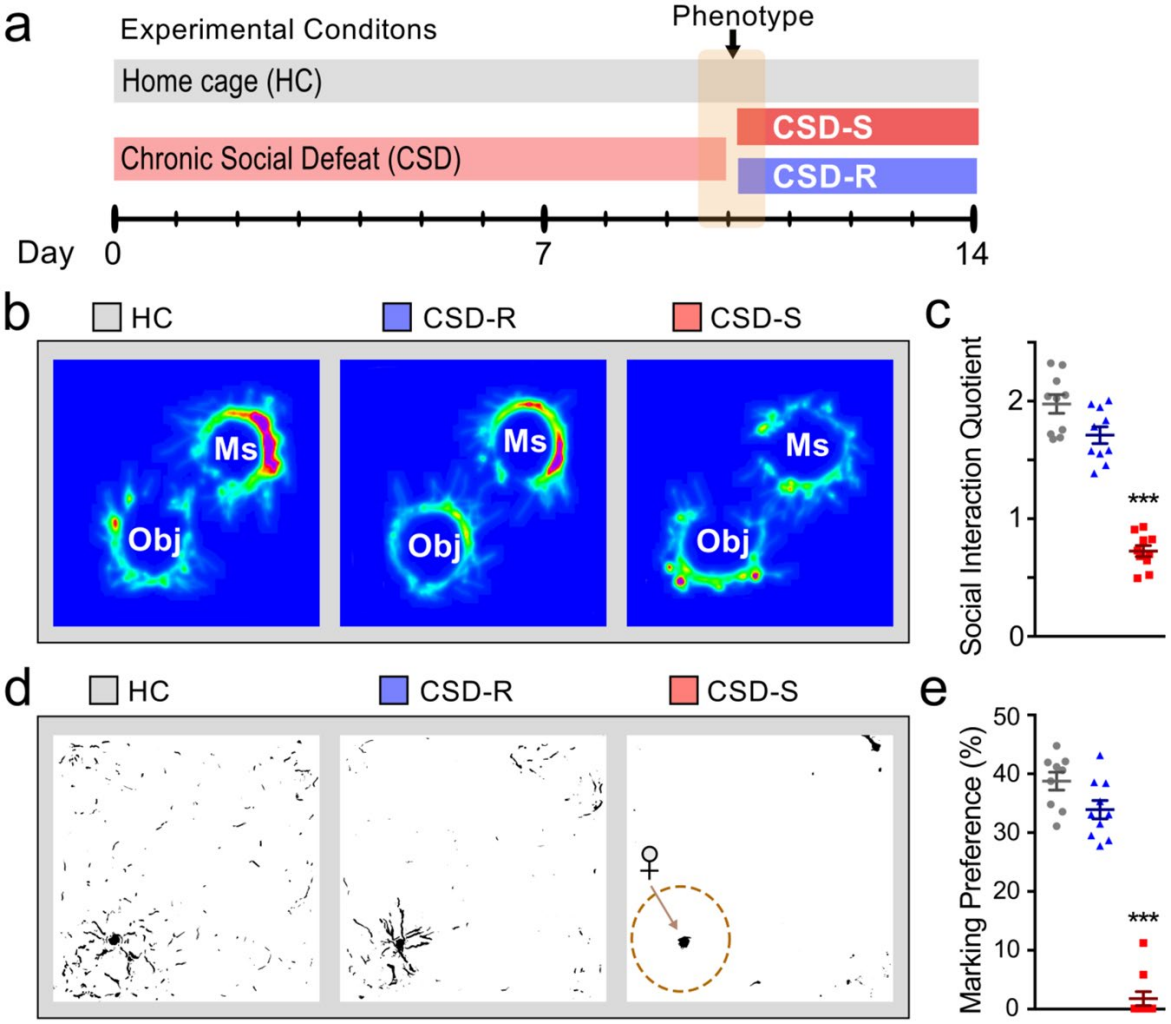
Chronic social defeat (CSD) results in susceptible (CSD-S) and resilient (CSD-R) behavioral phenotypes. (**a**) The experimental scheme. (**b–e**) The behaviors of the three groups tested on day 10 of the paradigm are shown for the social interaction task (**b**,**c**) and the urine scent marking task (**d**,**e**). Object (Obj) and mouse (Ms) stimuli were used to assess social preference in the SI task (**b**). One-way ANOVA followed by Bonferroni’s post hoc test (***p < 0.001 vs HC and CSD-R).

Most mice are behaviorally susceptible to chronic exposure to social defeat and show dramatic declines in social and territorial sexual behaviors. A subgroup of CSD mice however remains resilient and displays behavioral profiles comparable to nonstressed HC mice^23^. To stratify mice into CSD-S and CSD-R populations, on day 10 of the CSD procedure (Fig. 1a), we administered the social interaction (SI) and urine scent marking (USM) tests to measure sociability and hedonic drive. Declines in these social behaviors are maladaptive responses, and they coincide with anxiety-like and depressive-like behaviors measured in open field, light/dark box, elevated zero maze, sucrose preference test, forced swim test, and tail suspension test^17^. In mice chosen for microarray analysis, the CSD-S population showed significantly lower SI (*F*_(2,21)_ = 64.22, *p* < 0.0001) (Fig., 1b,c) and USM scores (*F*_(2,21)_ = 103.9, *p* < 0.0001) (Fig. 1d,e) compared to CSD-R and HC populations. CSD-R mice showed a behavioral profile similar to nonstressed HC controls (*p* > 0.05).

### Microarray analysis

**Figure 2:**
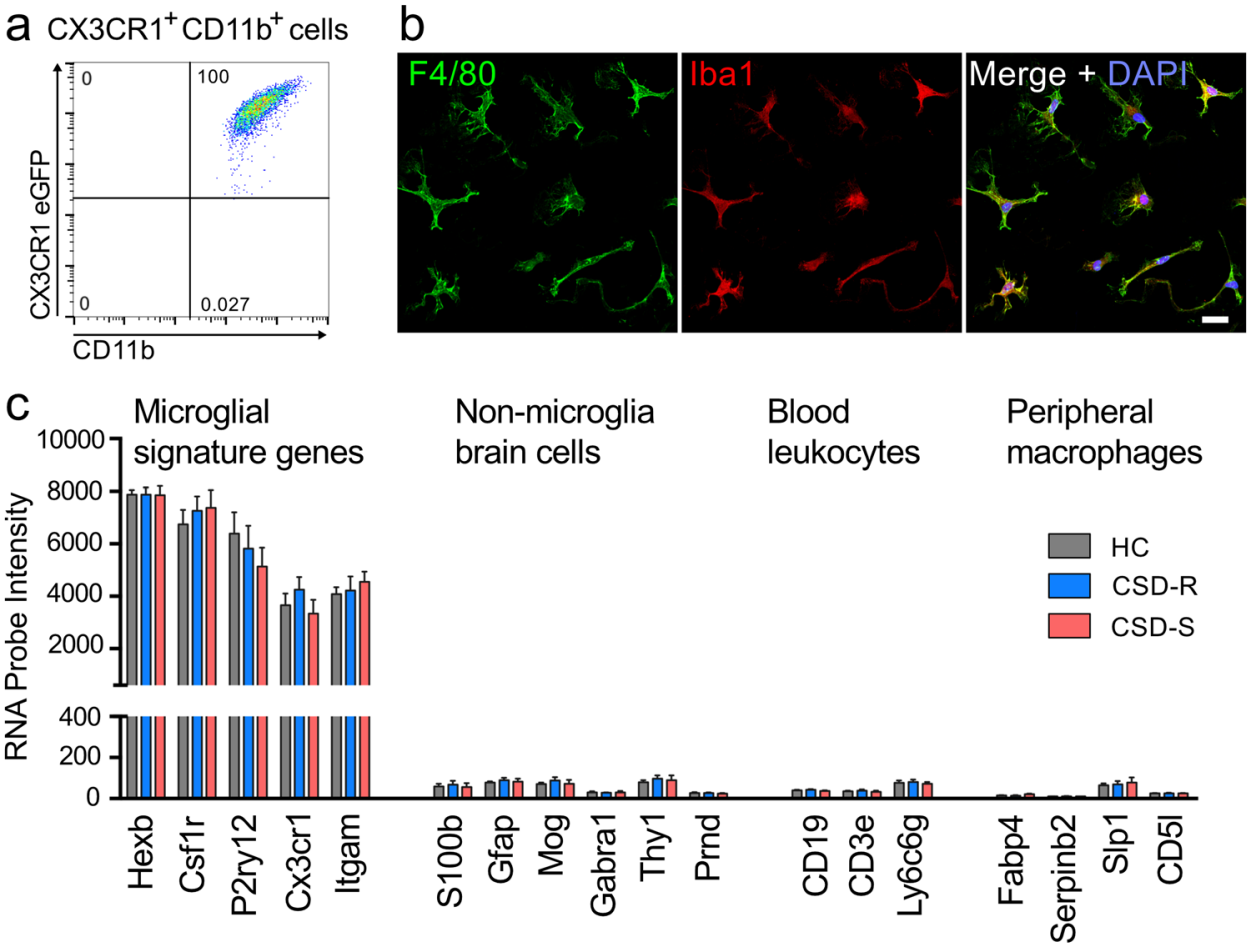
Extraction of microglia from *Cx3cr1*^*gfp/wt*^ mice by Percoll gradient and sorting for CD11b and GFP yielded a pure population of microglia. (**a**) Plot showing gating strategy and cell distribution. (**b**) Example of double immunostaining for microglial markers Iba1 and F4/80 in extracted cultured cells. Bar = 20 µm. (**c**) Gene expression profiles of cells extracted from the three groups of mice indicating that the cells are microglia and, notably, not myeloid cells of peripheral origin.

We asked if the activation state of resident microglia is dependent on the psychological status of the animal following chronic stress. We used microarray analysis to determine gene expression patterns in microglia from CSD-S, CSD-R, and HC mice to gain insight into brain dynamics responsible for differences in behavior between the groups. Microglia were purified using a combination of density gradients and immuno-magnetic separation for CD11b^hi^ cells. The consistency and purity of microglial extraction were pre-validated using *Cx3cr1*^gfp/wt^ reporter mice with green fluorescent microglial cells. Flow cytometry confirmed that the purified selected fraction (CD11b^hi^) showed near ubiquitous expression of GFP, and the purified non-selected fractions (CD11b^lo^) had no GFP^hi^ cells, which confirmed that CD11b is an efficient marker for microglia purification (Fig. 2a). Cells from purified CD11b^hi^ fractions from wildtype mice, all immunostained positively for the common microglial/macrophage antigens Iba1 and F4/80 (Fig. 2b). Gene expression profiles of purified CD11b^hi^ microglia subjected to microarray were highly enriched for established microglial signature genes including *Itgam*, *Csf1r*, *Cx3cr1*, *Hexb*, *and P2ry12*^24,25^. Signature markers for neurons, astrocytes, and oligodendrocytes were expressed at negligible levels. Finally, genes highly expressed in blood leukocyte subsets including C*d19* (B lymphocytes), *Cd3e* (T lymphocytes), *Ly6g* (granulocytes), and peripheral macrophages (*Fabp4*, *Serpinb2*, *Slpi*, and *Cd5l*), were not significantly evident in purified microglia (Fig. 2c). No differences in the expression levels of these signature genes were observed between microglia isolated from stressed and control mice. Combined, these data verified that our technique generated purified microglial populations.

**Figure 3:**
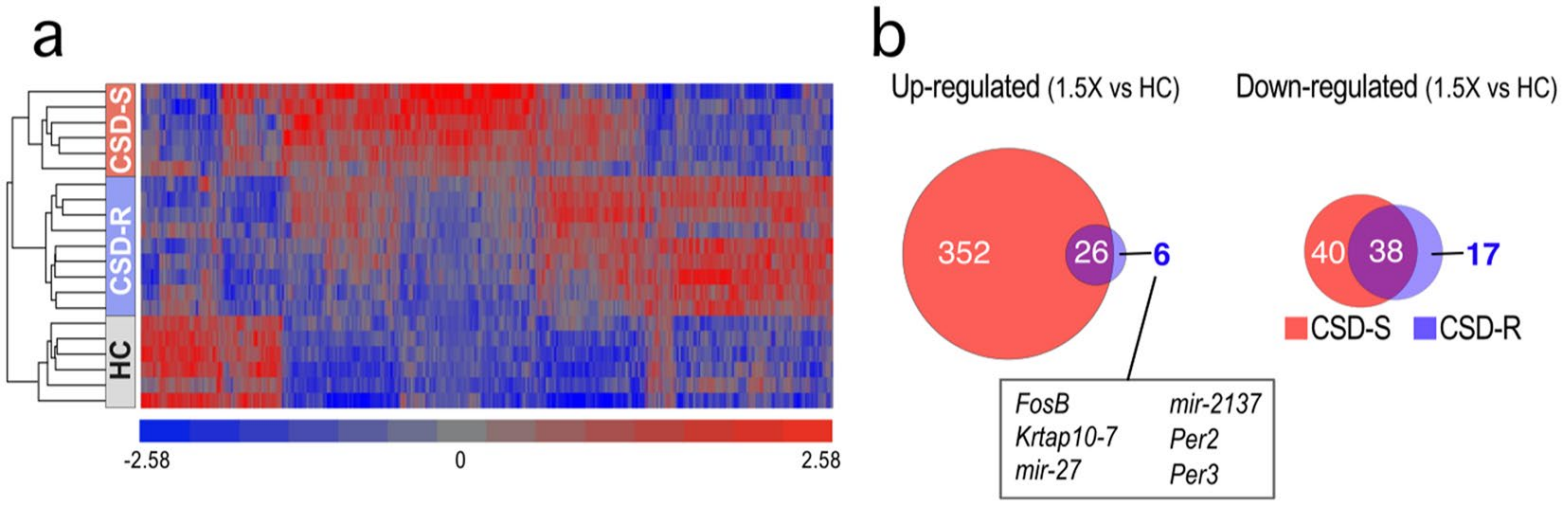
Microarray analysis of microglial gene expression in HC, CSD-S, and CSD-R mice shows a major effect in susceptible microglia. (**a**) Heat map results from unsupervised clustering for all differentially expressed genes reveals treatment grouping (One-way ANOVA; false discovery rate (FDR) *q* < 0.05). (**b**) Comparison of up- and down-regulated genes with ≥1.5x change in gene expression in the two defeated groups compared to HC.

Analysis of purified CSD-S, CSD-R, and HC microglia identified 2195 differentially expressed genes (One-way ANOVA; Partek, false discovery rate (FDR) *q* < 0.05). Hierarchical clustering demonstrated a marked contrast in expression profiles between microglia from CSD-S and CSD-R microglia (Fig. 3a), further illustrated by Venn diagrams (Fig. 3b) showing that social defeat altered gene expression in both CSD groups relative to HC, but the differences were far greater in the CSD-S group than the CSD-R group, especially in the up-regulated category.

### Bioinformatics

#### Network analysis

**Figure 4:**
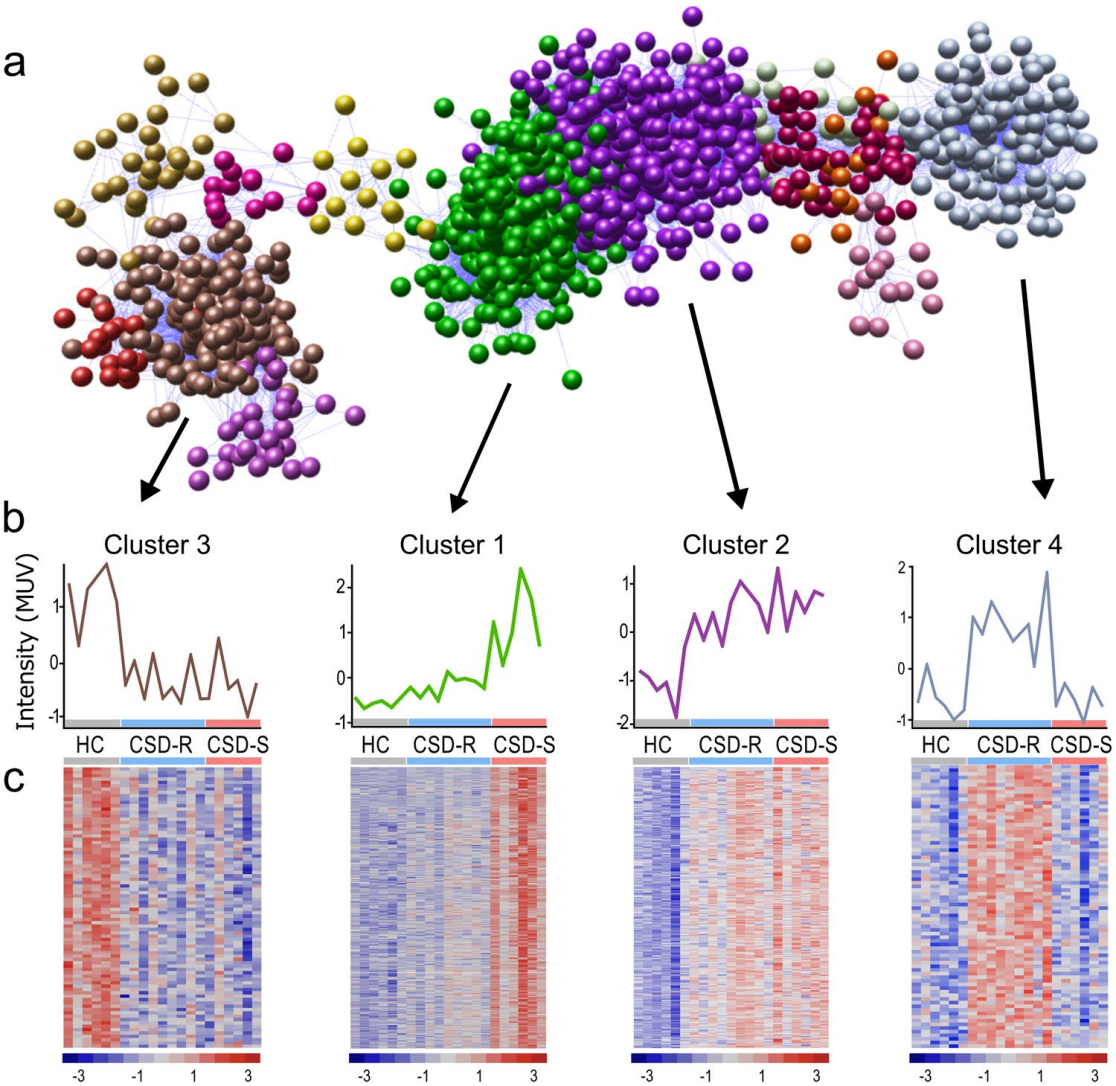
Distinct clusters of correlated, co-expressed genes emerge from network analysis of transcriptomes from stress-stratified microglia. (**a**) A transcript-to-transcript correlation network graph of transcripts significantly differentially expressed (DE) by social defeat was generated in Miru (Pearson correlation threshold *r* ≥ 0.80). Nodes represent gene transcripts, and edges represent the degree of correlation in co-expression. The network graph was clustered using a Markov clustering algorithm, and transcripts were assigned a color according to cluster membership. This resulted in 14 clusters containing >10 nodes. (**b**) Mean unit variation (MUV) profile of all transcripts that compose the four largest clusters. (**c**) Heat maps showing the expression profile of all transcripts contained within these clusters. Z-scores of individual probe sets are represented by color with red indicating high expression and blue indicating low expression.

We next used unsupervised clustering using the Miru network analysis tool to define patterns of gene coexpression that could characterize microglial phenotypes. In this approach, normalized gene expression data are transformed into a network graph where nodes in the graph represent transcripts connected to each other due to their coexpression across multiple samples. The similarity of a node’s expression signature with other nodes, or co-expression, determines its spatial location on the network graph. The networked 2195 differentially expressed transcripts were clustered using a Markov clustering algorithm to subdivide the graph into discrete sets of coexpressed genes, allowing unbiased identification of 14 gene clusters (Fig. 4a) that shared similar expression patterns. The clusters each contained 10–469 nodes (genes), and network topography revealed four major clusters that clearly distinguished the three conditions (Fig. 4b). Annotated lists of transcripts within clusters 1–4 are provided in Supplemental Table 1. The mean intensity profiles of these clusters showed that cluster 1 contained genes substantially elevated in CSD-S microglia only. Cluster 2 was defined by genes elevated in both stressed conditions. Cluster 3 was spatially more distant and contained genes whose expression was suppressed by stress. Cluster 4 contained genes selectively elevated in CSD-R microglia. Heat maps showed that the expression profile for individual genes within each cluster generally followed the cluster mean (Fig. 4c).

**Figure 5:**
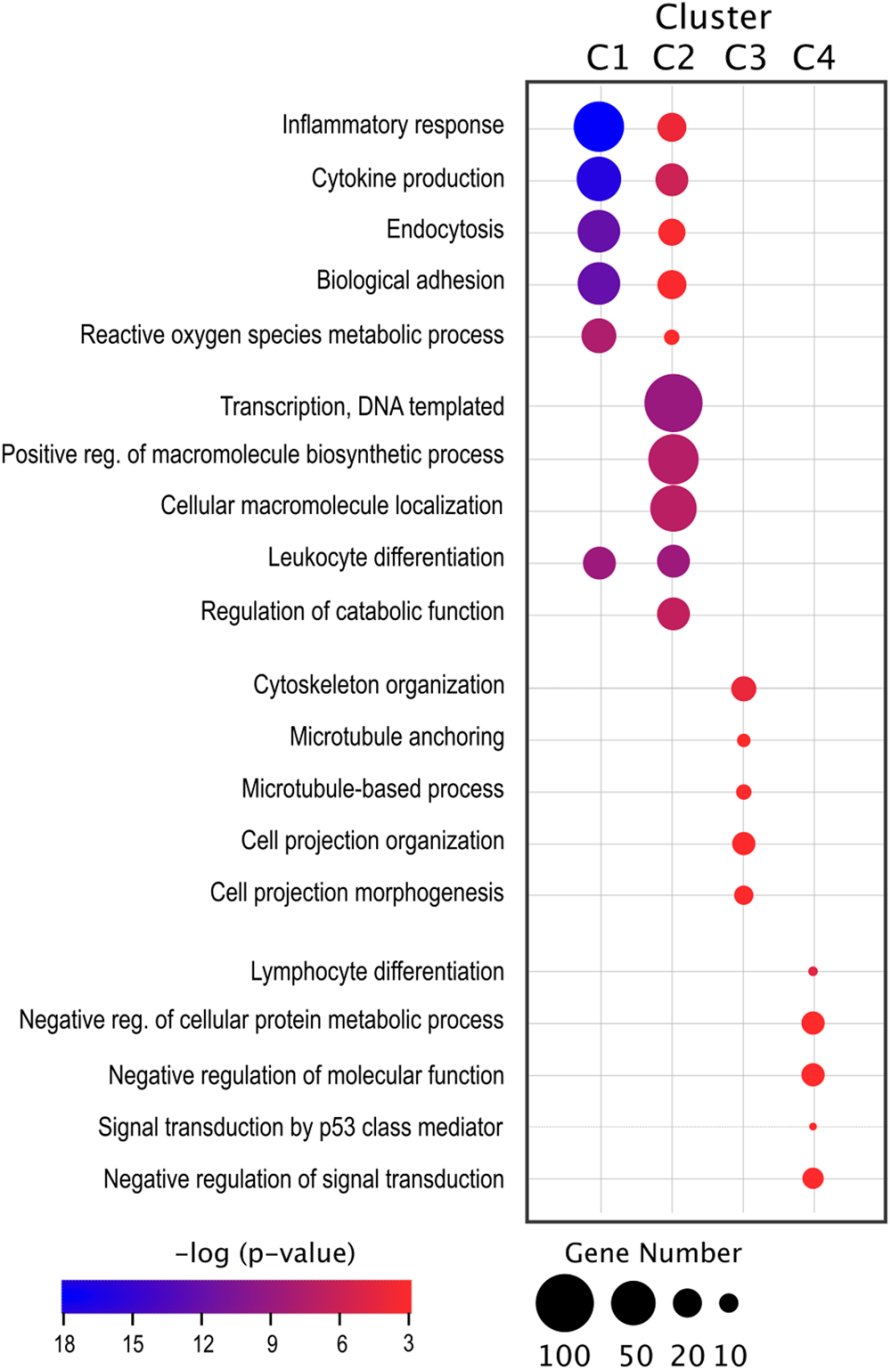
Gene Ontology (GO) enrichment analysis of gene clusters reveals divergent biological pathways between microglia from stress stratified mice. GO enrichment analysis for the four largest clusters was performed using DAVID. GO terms along with their *p*-values were further summarized by REViGO reduction analysis, removing redundant terms. The bubble chart shows the top five representative terms for each of the top four gene clusters. Bubble size indicates number of genes associated with each term. Bubble color indicates *p*-value significance.

Genes with linked functional activities or biological processes typically share analogous expression profiles^26^. We used DAVID to identify enriched biological process Gene Ontology (GO) functions within each cluster (fully annotated in Supplemental Table 2). Lists of enriched GO terms were further curated (interpreted/summarized) with REViGO, a clustering algorithm that relies on semantic similarity measures to remove redundant terms. This method provided a more precise interpretation of GO enrichment analysis; fully annotated lists provided in Supplemental Table 3. Among the top ranked representative biological processes in cluster 1 (high expression in CSD-S only) were immune-related processes including; ‘inflammatory response’, ‘cytokine production,’ ‘endocytosis,’ ‘leukocyte cell adhesion,’ and ‘ROS metabolic processes’ (Fig. 5). Among the genes in this cluster, many are involved in cell adhesion and motility pathways through interactions with matrix components and other extracellular ligands.

**Figure 6:**
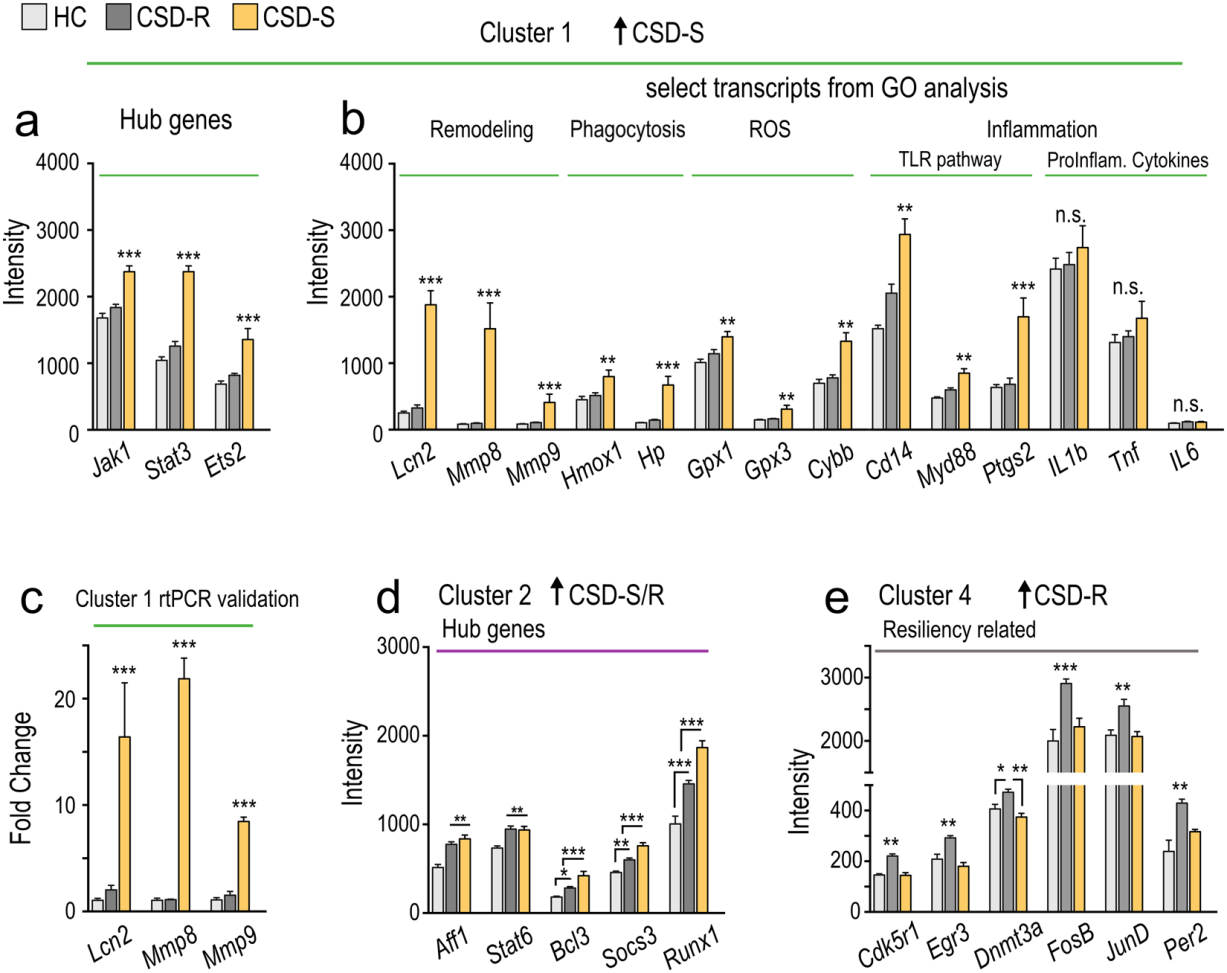
Expression levels of select transcripts identifying either hub genes or characterizing biological pathways divergent between microglia from stress stratified mice. (**a**) Mean expression intensity profile of hub genes in cluster 1 from the microarray. (**b**) microarray intensity profiles for elevated cluster 1 (green bars) genes grouped by GO analysis. (**c**) qPCR-derived mRNA expression levels of select cluster 1 genes involved with extracellular matrix (ECM) remodeling (*n* = 4/group). (**d**) microarray intensity profiles for elevated cluster 2 hub genes (magenta bar). (**e**) microarray intensity profiles for selected elevated cluster 4 genes (gray bar). Data show mean ± SD, *n* = 6 for HC and CSD-S, *n* = 9 for CSD-R. One-way ANOVA with Bonferroni correction (*p < 0.05, **p < 0.01, ***p < 0.001).

We interrogated the two largest clusters (1 and 2) to reveal highly interconnected genes in the co-expression network, where interconnectedness is indicative of hub nodes within each cluster. Hub nodes are frequently more relevant to the functionality of the network than other nodes and are important determinants of phenotype^27^. Our major focus of attention was cluster 1 (genes elevated selectively in CSDS-S); GO analysis revealed *Jak1*, *Stat3*, *and Ets2* as the most interconnected nodes in this cluster (Fig. 6a). Ingenuity Pathway Analysis (IPA) further confirmed these hub nodes as top upstream regulators (*z* scores >2.0). Importantly, within microglia and macrophages, these genes code for transcription factors that regulate extracellular matrix (ECM) remodeling, endocytosis/phagocytosis, ROS activity, and inflammation^28–30^, consistent with the immunomodulatory GO profile of cluster 1. Highly significant, differentially expressed genes from the array related to these biological pathways were more closely examined. Three ECM remodeling genes—*Lcn2*, *Mmp8*, *and Mmp9—*showing particularly large elevations in expression within the CSD-S group (Fig. 6b) were subjected to qPCR analysis (Fig. 6c), which confirmed the microarray data.

Cluster 2 (high expression in both CSD-S and CSD-R) was dominated by metabolic and macromolecular biosynthesis processes (Fig. 5). Hub genes *Aff1* and S*tat6* were similarly elevated in both stress subgroups (Fig. 6d) and were revealed as upstream regulators through IPA examination. These transcriptional regulators are linked to the metabolic reprogramming of macrophages towards alternative activation states^31,32^ and support the metabolic gene profile of cluster 2. *Bcl3*, *Socs3*, and *Runx1* were other highly interconnected hub genes in cluster 2 (Fig. 6d), but the expression pattern was substantially elevated in CSD-S versus CSD-R. These regulators act to attenuate cytokine signaling cascades^33–35^, and the prevalence of these regulators suggests microglia in CSD-S brain but not CSD-R brain are responding to proinflammatory stimuli. Full annotation of nodes within clusters 1 and 2 is provided in Supplemental Table 4.

Microglia rapidly alter their structure and morphology in response to activation signals. Therefore, we were interested to see that cluster 3 (suppressed expression in CSD-S and CSD-R) contained a large number of genes associated with cell morphology. GO analysis revealed ‘cytoskeletal organization,’ ‘cell projection organization,’ and ‘microtubule-based process’ as among the most overrepresented processes (Fig. 5). Suppression of these biological processes may allow microglia to exit their active surveillance state, retract processes, and perhaps move through brain parenchyma towards sites of CNS injury.

Cluster 4 (Fig. 6d) contained genes expressed at relatively greater levels in microglia from CSD-R versus HC or CSD-S mice. Among the small number of genes in this cluster, many (e.g., *Cdk5r1*, *Egr3*, *Dnmt3a*, *Fosb*, *JunD*, and *Per2*) are involved in regulating cellular and behavioral plasticity to emotional stimuli^7,36–39^. Of particular note, *Dnmt3a* and *Fosb* are directly implicated as essential mechanisms of resilience in mice^7,36^. GO analysis showed this cluster was populated by terms describing a decline in cellular activity including negative regulation of cellular protein metabolic process, molecular function, and signal transduction (Fig. 5). This may reflect disengagement of resilient microglia from biological programs that drive activation states characterized by susceptible cells.

#### Gene Set Enrichment Analysis (GSEA)

**Figure 7:**
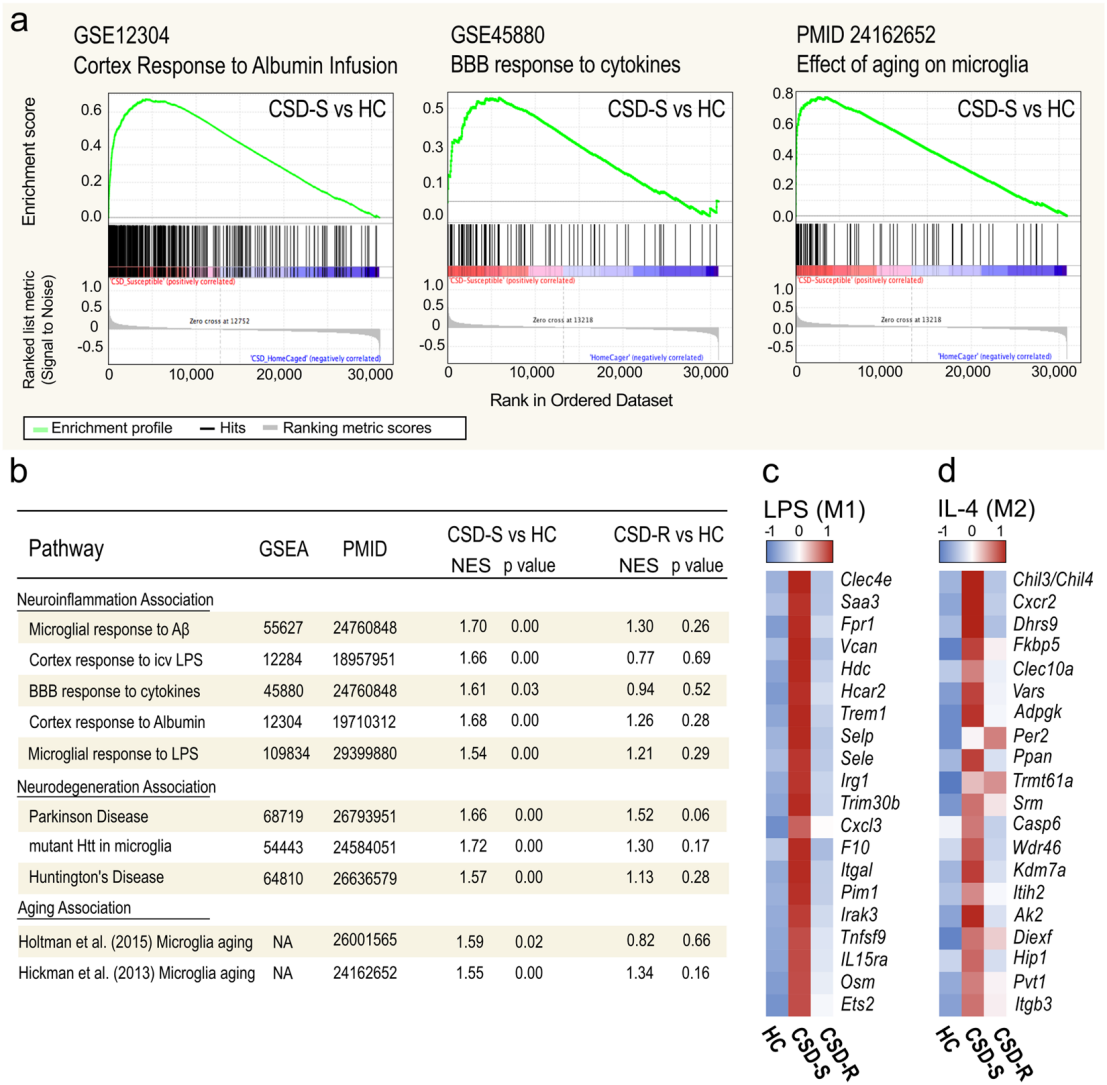
Microglia from CSD-S mice have expression profile seen in inflammation, neurodegenerative disease, aging, and response to albumin. Microarray data were analyzed using gene set enrichment analysis (GSEA) software to identify functionally related gene sets with statistically significant enrichment in stressed microglia compared with several neuroinflammatory models. (**a**) Plots show GSEA enrichment in array data from CSD-S microglia compared with Cortex response to albumin infusion^93^, BBB response to cytokines^75^, and elevation in aging^44^ as the expression phenotype. The plots show the distribution of genes in each set that are positively and negatively correlated with the respective responses. (**b**) Gene sets from CSD-S but not CSD-R microglia highly correlate with gene sets from several models of neuroinflammation^75,93–96^, neurodegeneration^40–42^, and aging^44,74^.(**c**,**d**) Heat maps detailing the microarray expression patterns of unique LPS-induced (**c**) and IL-4 induced (**d**) genes^46^ that were differentially expressed across HC, CSD-S, and CSD-R microglia. Row- z-score intensity was used to represent expression differences across groups. NES, normalized enrichment score; H, HC; R, CSD-R; S, CSD-S. p-value, nominal p value.

We next used GSEA to search other gene expression databases derived from either inflammatory manipulations or diseases that might share expression profiles similar to that found in CSD-S microglia. Genes upregulated in CSD-S microglia were markedly correlated with genes up-regulated by administration to brain of known neuroinflammatory compounds including amyloid β, lipopolysaccharide (LPS), TNFα, and albumin (Fig. 7a,b). At the disease level, genes upregulated in CSD-S microglia were strikingly associated with genes upregulated in both human Parkinson’s or Huntington’s disease brains (GSEA68719, GSEA64810)^40,41^. The transcriptional signature of CSD-S microglia was also strongly associated with mouse microglia that express mutant forms of Huntingtin (*Htt*) (GSEA54443)^42^. Within these enriched gene sets, several transcripts involved with erythrophagocytosis and hemoglobin scavenging were detected, including Heme oxygenase1 (*Hmox1*) and Haptoglobin (*Hp*), suggesting that these cells may be exposed to plasma proteins that are normally excluded by the BBB^43^. No significant correlations were detected with CSD-R samples. Aging has substantial effects on the microglia transcriptome^44^, and chronic stress is thought to accelarate brain aging^45^. We observed that the profile of CSD-S microglia is strikingly similar to profiles reported for aged microglia (Fig. 7a,b). No significant correlations were detected with CSD-R samples.

We wondered if the immunophenotypic identity of CSD-S and CSD-R microglia could resemble activation states of explicitly polarized microglia. Drawing from published microglia microarray data^46^, we determined a set of 303 non-overlapping genes significantly upregulated by the M1 polarizing agent LPS (>5-fold, FDR *q* < 0.05) and 227 genes unique to the M2 polarizing agent IL-4 (>1.5-fold, FDR *q* < 0.05). These gene lists were overlaid on our data using GSEA, revealing 115 LPS-induced and 38 IL-4-induced genes significantly enriched in our data set. The top 20 most enriched transcripts are shown in Fig. 7c and d, which reveals concomitant elevated expression of both M1-like (M_LPS_) (Fig. 7c) and M2-like (M_IL4_) (Fig. 7d) genes in CSD-S microglia. Canonical markers of divergent polarization states—*Nos2*, *Tnf*, and *Arg1*^46^—were notably absent from these lists. These data point to the inadequacy of assigning binary polarization states to microglia.

### Functional Assays

#### BBB permeability

**Figure 8:**
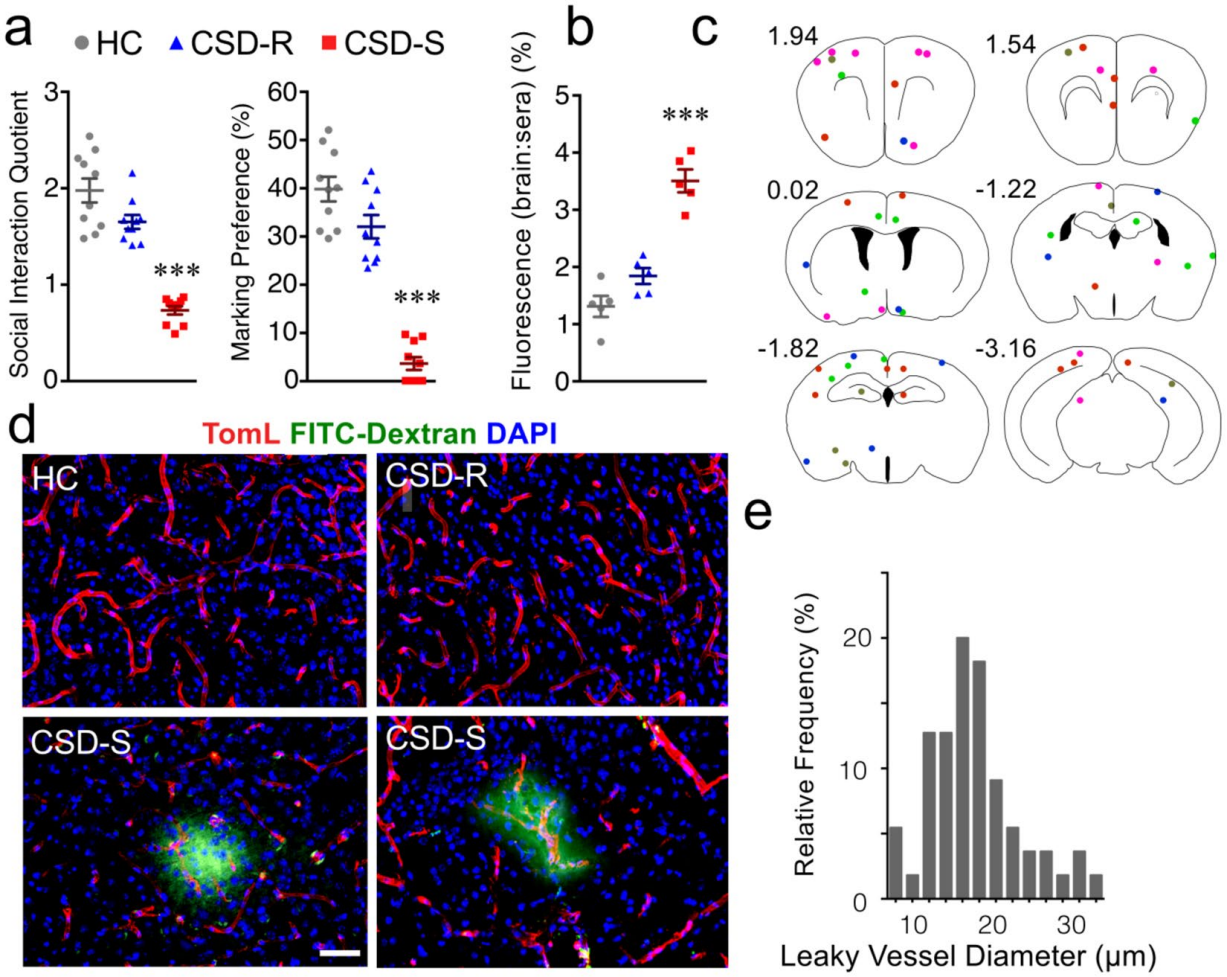
The blood-brain barrier is compromised by stress selectively in CSD-S mice. (**a**) A new set of animals was screened for susceptibility or resilience (*n* = 10/group). (**b**) Penetration of the fluorescent tracer sodium fluorescein (*n* = 5/group). (**c**) Anatomical locations of microbleeds indicated by Alexa Fluor 488-dextran entry into the parenchyma. The dots are color-coded for individual mice in the CSD-S group (*n* = 5), bleeds were not detected in brains of HC or CSD-R mice. Number coordinates indicate distance from Bregma. (**d**) Examples of Tomato Lectin-stained vasculature for the three conditions, and isolated bleeds in CSD-S brain. Scale bar = 50 µm for all images. (**e**) Relative frequency of bleeds as a function of vessel diameter. One-way ANOVA followed by Bonferroni’s post hoc test (***p < 0.001 vs. HC and CSD-R).

Microglia from CSD-S mice selectively express transcripts involved with collagen degradation and ECM breakdown, including *Mmp8*, *Mmp9*, and *Lcn2* (Fig. 6b,c), suggesting this stress subgroup may have changes in vascular structure^47^. We behaviorally stratified a fresh cohort of CSD mice (Fig. 8a) and evaluated BBB permeability with intravenously injected tracers. After i.v. injection of sodium fluorescein (0.376 kDa), there was a significantly elevated brain:serum ratio of fluorescence in CSD-S mice compared to all other groups, suggesting greater leakage of fluorescein across the BBB within this cohort (*F*_(2,17)_ = 25.5, *p* < 0.001) (Fig. 8b).

Next, we intravenously administered a larger molecular weight (10 kDa) fixable Alexa Fluor 488-dextran tracer and detected extravascular dextran by microscopy in the parenchyma of CSD-S but not HC or CSD-R brains (Fig. 8c,d). The dextran leaks were sporadically distributed throughout the brain, but they were primarily observed in cortical regions (Fig. 8c). This is in contrast to a recent report of CSD-induced vascular pathology in stress-susceptible mice that was confined to the ventral striatum^48^. When cerebral blood vessels associated with the dextran leaks were clearly distinguishable, their internal diameter was found to be in the range of ∼10–20 µm (Fig. 8e), the normal range of size for arterioles that are the source of cortical microinfarcts^49^. White matter remained essentially impermeable to dextran leaks.

#### Phagocytic activity

**Figure 9:**
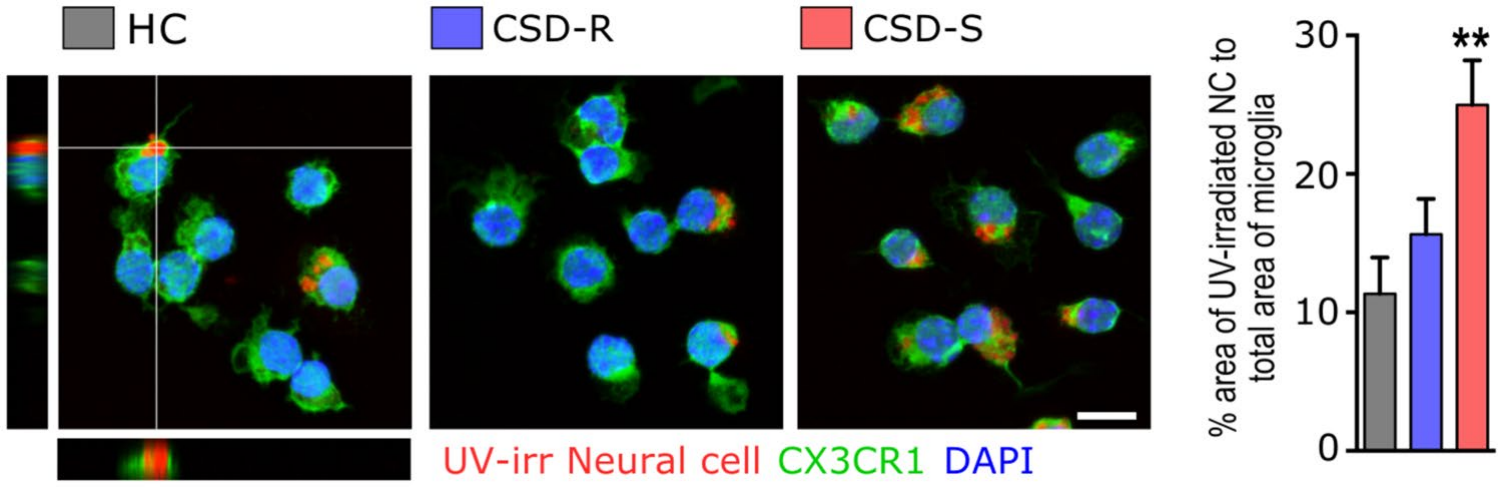
*Ex vivo* phagocytosis of labeled neuronal debris is elevated in CSD-S microglia. Microglia were co-cultured with TAMRA-labeled neuronal stem cells exposed to UV light. The total area of phagocytosed labeled material was compared to the microglia area and used as an index of phagocytic capacity. Phagocytic index is shown (*n* = 7/group). Scale bar = 10 µm. One-way ANOVA followed by Bonferroni’s post hoc test (**p < 0.01 vs. HC and CSD-R).

We confirmed further whether phagocytic activity was elevated in CSD-S microglia using a functional *ex-vivo* assay. Here, recently isolated microglia were seeded with pre-labeled UV-irradiated neural cells, used as a source of apoptotic debris. Microglia from CSD-S brains were observed to have phagocytosed more labeled material compared to all other conditions (Fig. 9) (*F*_(2,20)_ = 37.6, *p* < 0.001).

#### ROS activity

Gene expression of enzymes important for countering oxygen radicals produced during oxidative phosphorylation, including glutathione peroxidase (*Gpx1* and *Gpx3*), was elevated in CSD-S microglia (Fig. 6b), suggesting altered cellular redox activity. We evaluated this finding with visualization of dihydroethidium (DHE), a membrane-permeable dye that indicates respiratory burst activity^50^ and enables quantification of *in-vivo* ROS production^51^.

**Figure 10:**
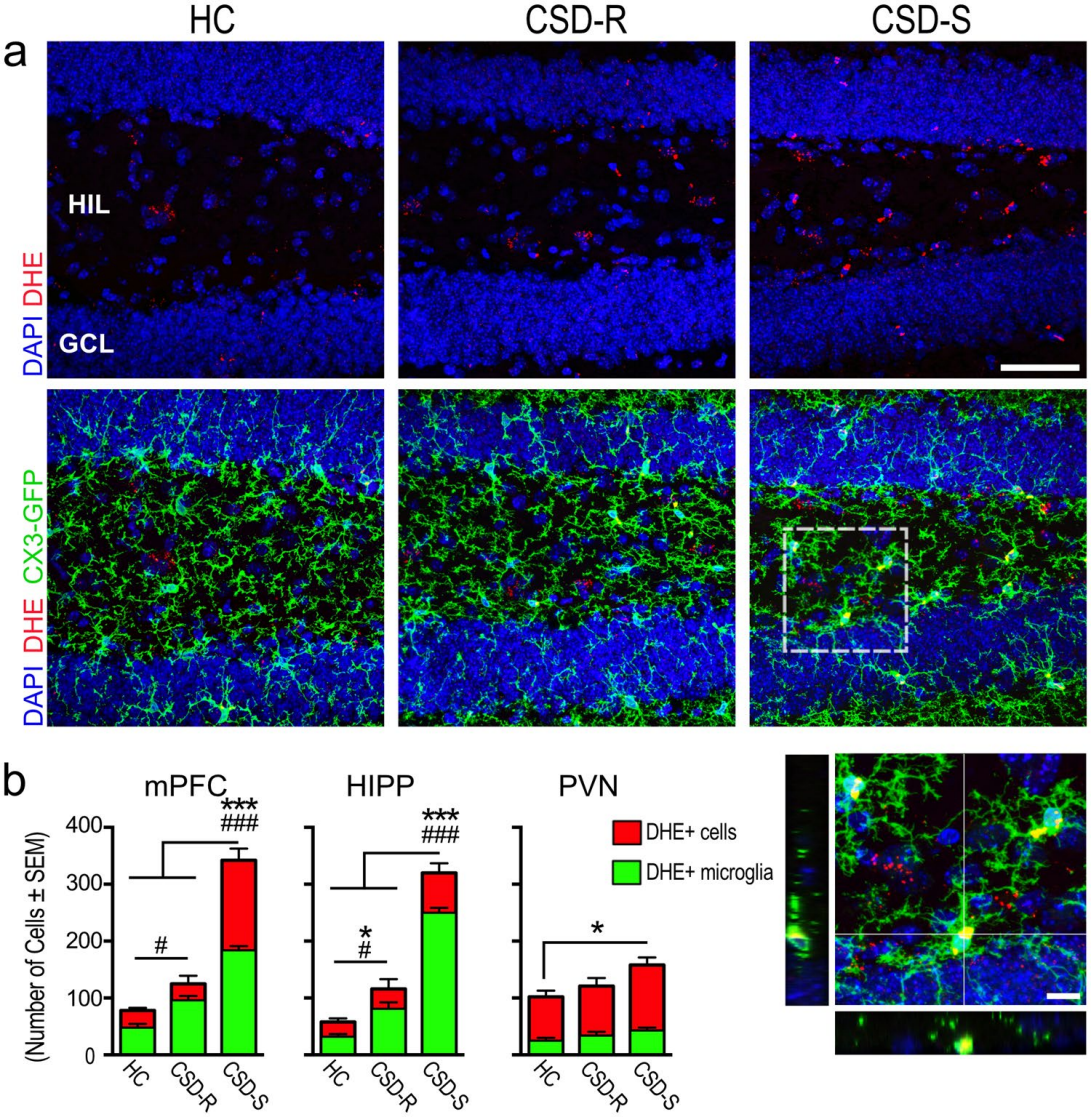
Oxidative stress occurred in the CSD-S group in the form of elevated ROS revealed by DHE staining. (**a**) Examples of DHE cellular localization in *Cx3cr1*^*gfp/wt*^ mice, with the box magnified and shown in the x, y, and z planes in the lower right. (**b**) Numbers of DHE-labeled cells and their extent of colocalization with GFP-positive microglia. One-way ANOVA followed by Bonferroni’s post hoc test was used for each analysis in each area. Bonferroni correction for multiple comparisons has been applied (DHE+ cells; *p < 0.05, ***p < 0.001) (DHE+ microglia; ^#^*p* < 0.05, ^###^*p* < 0.001) (n = 6/group). Magnification bars = 50 µm in (**a**) and 10 µm in inset.

We examined DHE labeling in a fresh cohort of behaviorally phenotyped *Cx3cr1*^*gfp/wt*^ mice, and focused on three brain regions known to show altered function in patients suffering from affective disorders^52^ and altered neuronal activity in mice in the CSD paradigm^53^. DHE labeling in the medial prefrontal cortex (mPFC) and hippocampus (Fig. 10a) was significantly elevated in CSD-S mice compared to HC and CSD-R mice (HIPP; *F*_(2,17)_ = 89.9, *p* < 0.001, mPFC; *F*_(2,17)_ = 92.8, *p* < 0.001). Small but significant elevations in DHE labeling were detected in the hypothalamic paraventricular nucleus of CSD-S mice, whereas no change was observed in CSD-R mice compared to HC (*F*_(2,17)_ = 4.8, *p* < 0.025).

Microglial sources of DHE labeling were identified by colocalization with GFP in *Cx3cr1*^*gfp/wt*^ mice^54^, which provide clear identification of microglia for the purpose of analysis. In the mPFC and hippocampus, microglia were the chief source of DHE labeling in stressed mice in both subgroups (HIPP; *F*_(2,17)_ = 174.3, *p* < 0.001, mPFC; *F*_(2,17)_ = 91.0, *p* < 0.001). CSD-S mice had significantly more DHE-positive microglia in the mPFC and hippocampus compared to all other groups. CSD-R mice also showed significantly more DHE microglia in these areas compared to HC, although the effect was substantially diminished (Fig. 10b). No effect of stress on PVN DHE-positive microglia was detected.

#### No extravasation of blood leukocytes

**Figure 11:**
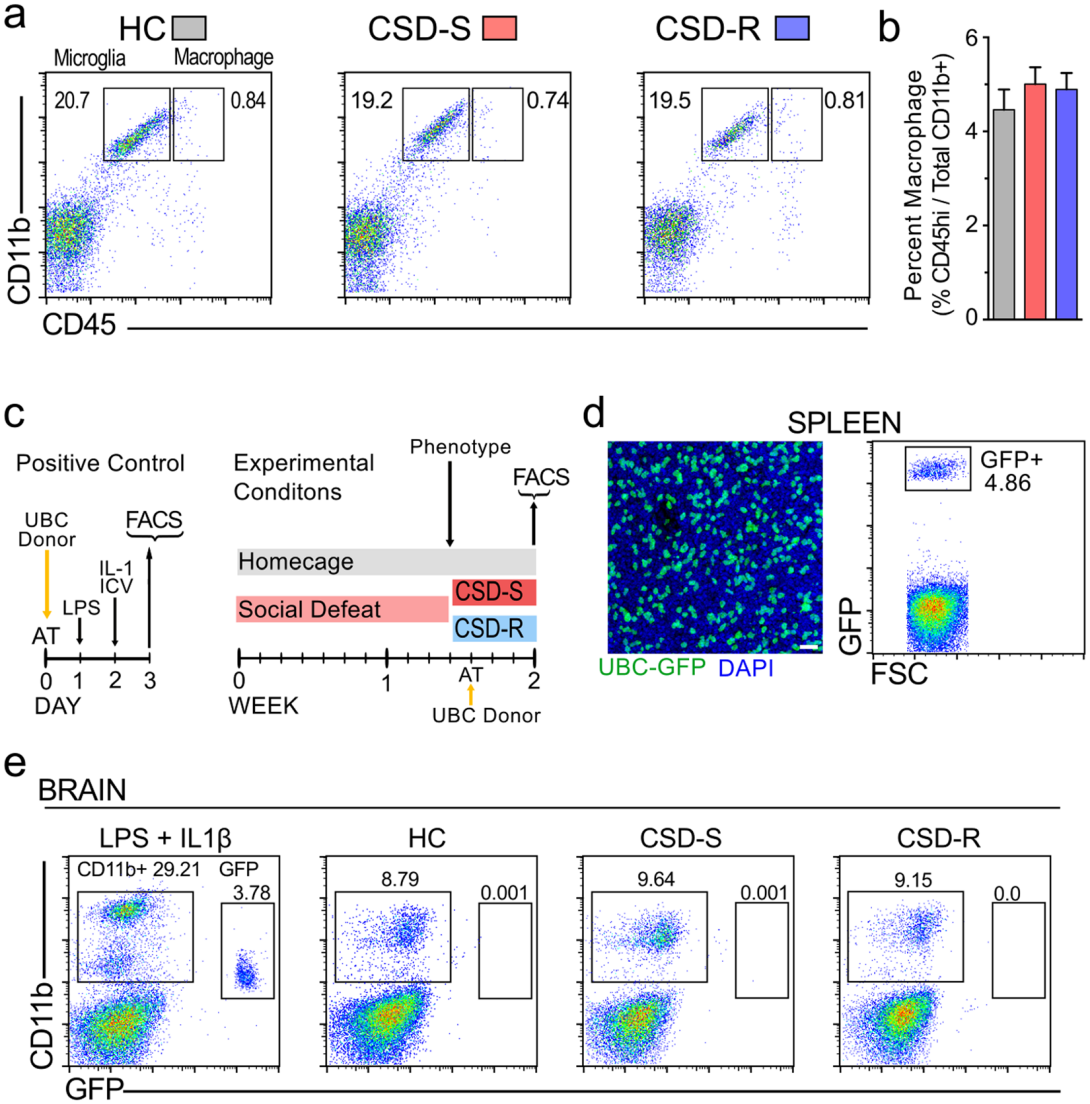
Social defeat did not induce infiltration of leukocytes into brain. (**a**) Representative bivariate dot plots show brain cells from HC, CSD-S, or CSD-R mice stained and gated for microglia (CD11b^hi^ CD45^low^) and peripheral leukocytes (CD11b^hi^ CD45^hi^). (**b**) Number of leukocytes in the brain was similar between all treatment conditions. Results are expressed as percentage CD45^hi^ cells in the CD11b^hi^ cell population (*n* = 6 per group). One-way ANOVA detected no significance between groups. (**c**–**e**) GFP-positive splenocytes transferred from *Ubc*^*gfp/gfp*^ mice extravasate into the brain of wildtype mice challenged with central plus peripheral inflammagen, but not in stressed mice. (**c**) The design of the experiments. AT, adoptive transfer of gradient-purified spleen cells from a *Ubc*^*gfp/gfp*^ donor mouse. FACS, flow cytometry. (**d**) The successful transfer of GFP-labeled leukocytes in the spleen of a HC mouse is shown by immunofluorescence staining of spleen sections and by flow cytometry. Magnification bar = 20 µm. (**e**) GFP-labeled leukocytes were present in the brain after immune challenge but not after CSD stress. (*n* = 4 per group).

Many of the gene transcripts elevated in CSD-S microglia are involved in cell adhesion, motility, and leukocyte extravasation. We used flow cytometry in a fresh cohort of behaviorally-phenotyped defeated mice to reveal if peripheral leukocytes migrate into brain of CSD-S and CSD-R mice. Resident microglia were differentiated from CNS-infiltrating leukocytes based on expression level of CD11b and CD45. In wildtype mice, brain microglia and leukocytes were collected 2 h after the final social defeat. Figure 11a shows representative plots of CD11b^hi^ CD45^lo^ microglia and CD11b^hi^ CD45^hi^ leukocytes, including monocyte/macrophages^55^. Neither CSD-S nor CSD-R brains showed elevated numbers of CD45^hi^ leukocytes relative to HC brains (Fig. 11b, *p* = 0.57).

It is possible that CNS-infiltrating leukocytes lose their identifying expression of extracellular markers once in the brain microenvironment and become indistinguishable from resident microglia. To address this concern, spleen cells from *Ubc*^*gfp/gfp*^ mice with ubiquitous cellular expression of GFP were adoptively transferred into wildtype mice after phenotyping (Fig. 11c) to determine by another means if peripheral leukocytes are recruited into brain. Indeed, a robust recruitment of transferred GFP-positive cells into the brain was detected in the positive control group following immune stimulation (Fig. 11e). However, in accordance with our previously published results^17^, GFP-positive cells were detected in the spleen after adoptive transfer (Fig. 11d), but they were not detected in brains of stressed mice, neither in the CSD-S nor CSD-R brains (Fig. 11e).

## Discussion

CNS neuroinflammation has been proposed as a key etiopathological mechanism underpinning a subset of mood disorders^56,57^. Microglia are the key resident immune cells in brain and are a major contributor to neuroinflammation. These cells are in constant surveillance mode and are therefore uniquely situated biosensors for stress effects on the CNS microenvironment. Our goal was to decode microglia responses to chronic psychological stress and reveal physiological mechanisms that could drive resiliency and susceptibility to stress-induced psychopathology.

We performed transcriptome analysis of microglia isolated from whole brains of HC and chronically stressed mice stratified into stress-susceptible (CSD-S) and -resilient (CSD-R) subgroups. Strikingly, though mice in both subgroups underwent social defeat and were rendered subordinate to the aggressor mouse, significant differences existed in gene expression profiles, with the CSD-S microglia differing on a number of gene ontology and functional pathway scales contrasted with fewer and more subtle changes in the CSD-R microglia relative to HC. Gene co-expression network analysis and GSEA revealed both functional differences and commonalities between stress subgroups. Major findings from bioinformatic (network and enrichment) analyses were that (1) immune-, phagosome-, and ROS-generation pathways contribute prominently to the phenotype of CSD-S microglia, (2) BBB dysregulation is a key driver of the CSD-S microglial transcriptome profile, (3) the CSD-S profile was strikingly similar to profiles in chronic neurodegenerative conditions, (4) transcriptional networks controlling microglial metabolic and bioenergetic processes are key features in stressed microglia from both subgroups, and (5) genes implicated in countering stress were enriched in CSD-R microglia. The causal nature of the relationship between microglial reactivity and affective control over stress remains to be determined. However, we can speculate about the consequences of different physiological programs played out in susceptible versus resilient animals.

Pursuant to pathways within Cluster 1 (Figs 5 and 6), histochemical and functional assays confirmed the predicted increase in phagocytic activity, ECM remodeling, BBB leakage, and elevation in ROS activity in CSD-S mice. However, there was no evidence of leukocyte infiltration into the brain in any group. It appears that leukocyte extravasation may require additional factors, such as those associated with disease or injury, e.g., experimental allergic encephalomyelitis (EAE)^58^, liver injury^59^, or repeated long-lasting defeats accompanied by physical injury^60^. Although both resilient and susceptible mice were exposed to stress, the injury and inflammatory pathways prevalent in CSD-S microglia were not active in CSD-R microglia. BBB breakdown, predicted from the microglial expression profile, was present only in CSD-S mice, suggesting an important role played by microglia in this process.

CSD-S microglia were more actively phagocytic *ex vivo* than CSD-R or HC microglia. The leakage of blood components into susceptible brain coupled with the selective elevation of transcripts such as heme-oxygenase 1 (*Hmox1*/ HO-1) and haptoglobin (*Hb*) suggests one source of *in vivo* phagocytic cargo may be extravasated erythrocytes^61,62^. Other types of cargo, including myelin^63^ (which is reduced by CSD^64^) and material from synapse pruning and remodeling events^21^ (also prevalent during CSD) might be phagocytosed.

The removal of debris is generally a beneficial event but does result in several downstream consequences including respiratory burst activation and ROS generation^50^. CSD-S microglia were substantially enriched for pathways that lead to elevated ROS activity, and we confirmed the functional presence of these pathways using *in vivo* labeling of ROS production with DHE. As expected, DHE-positive microglia were markedly elevated in CSD-S animals. Microscopic examination of the labeling (Fig. 10a) revealed two putative intracellular sources of ROS production. First, a diffusely distributed punctate labeling surrounded the nucleus, possibly from mitochondrial sources. This was present in multiple cell types including neurons. Ambient levels of ROS production are important for mitochondrial homeostasis, signaling pathways, and metabolic activity^65^. Thus, it is reasonable to assume some level of ROS activity will occur absent of stress load. Indeed, even nonstressed brains showed low levels of DHE staining. A second pattern of DHE labeling, unique to CSD-S microglia, was also observed. Contrasted with punctate labeling, these larger blebs may represent phagosomal ROS generation involved in the degradation of engulfed cargo^66,67^. The elevation of blood products in CSD-S brain would create a microenvironment favorable to phagosome generation and ROS production in phagocytic cells^50^.

Prolonged or chronic ROS production is central to the progression of inflammatory disease and can lead to neuronal damage, BBB disruption through various intermediaries^68^, and perpetuation of the stress response. The CSD-S effect was observed across multiple measures including elevated transcriptional expression of integrins, immunoglobulin superfamily members, and scavenger receptors, which are generally associated with immune, phagosome, and ROS pathways^69^. These pathways are elevated in chronic neurodegenerative diseases and aging (Fig. 7b).

Our network-based integrative analysis highlighted the inflammation, phagocytosis, and ROS generation as genetic programs most strongly associated with the pathophysiology of CSD-S microglia that did not occur in CSD-R microglia. It also identified *Ets2*, *Jak1*, and *Stat3* as key cluster 1 hub genes regulating these biological processes (Fig. 6a). ETS2 functions as a modulator and amplifier of inflammatory response and promotes vascular leakage, possibly through the elevation of MMPs^70^. The Janus kinase (JAK)/signal transducer and activator of transcription (STAT) pathway is a well-characterized cellular signaling pathways that plays an essential role in promoting and modulating immune and inflammatory processes. Activation of the JAK/STAT pathway has been associated with pathological conditions such as cerebral ischemia, traumatic brain injury, and brain inflammation^68^ (review^29^;). Within CSD-S microglia, the increase in multiple transcripts involved with the TLR-signaling pathway, including *Cd14*, *MyD88*, *Irak4*, *Nfkb1*, and *Cox2* (Fig. 6) suggests the maintenance of a chronic, proinflammatory condition. These pathways are hallmarks of classically activated (M1) macrophages. However, the lack of transcriptional elevation for *IL-1β*, *TNFα*, *IFNγ*, *CSF1*, and *IL-6*, all well-defined proinflammatory cytokines, suggests an atypical inflammatory response^71^. In fact, some of the most highly altered genes in CSD-S microglia fall within the alternative activation (M2), wound resolution, camp shown in Fig. 7d. The stochastic occurrence of vascular leakages and chronic nature of the stress suggest microglial microenvironments that contain different stages of inflammation or resolution occurring continuously during CSD.

Importantly, the expression of these transcripts was not altered in CSD-R microglia, suggesting that physiological events that cause their expression in susceptible animals do not occur in resilient animals. Although we are not clear about the drivers of CSD-S phenotypes, BBB vascular leakiness occurs sporadically throughout brain only in CSD-S animals. GSEA data confirm that BBB leakiness is a key driver of the CSD-S microglial phenotype. We note the similarity with ‘Cortex Response to Albumin Infusion’ (Fig. 7a). A causal relationship between the highly correlated stress susceptibility and BBB disruption has not been determined. In clinical studies, risk factors such as hypertension^72^ and peripheral inflammation^73^, which occurs in CSD-S animals^5^, predict the occurrence of cerebral microbleeds. CSD-S microglia share a gene expression profile with brains of animals given peripheral lipopolysaccharide (LPS) challenge^74^, a model of peripheral inflammation (Fig. 7b). Blood-borne immune challenges, including inflammatory cytokines^75^ and activated leukocytes^76^, act on endothelial cells to compromise the BBB. It is likely that microbleeds create local environments that change as the wounds progress from inflammatory to remodeling. The spatial and temporal features would cause microglia in the vicinity to be differently programmed according to dynamic functional requirements (phagocytosis, ECM remodeling, and vascular repair). The observed features of CSD-S microglia support a close relationship between stress susceptibility and peripheral indices of inflammation and hypertension, meriting further study.

Cluster 1 genes and pathways have been a major focus thus far, but cluster 2 genes (upregulated in both CSD-S and CSD-R relative to HC) reveal transcriptional programs controlling transcriptional activity, catabolism, and macromolecular biosynthesis (Fig. 5), indicating increased metabolism and energy demand in stress-exposed cells independent of the behavioral outcome in the animals. It is fascinating to appreciate that the brain environment is challenged like this in a chronic stress situation, and microglia are actively participating in the process regardless of outcome. Further insight into this observation is provided by the set of pathways participating in cluster 4—genes upregulated only in CSD-R microglia—indicating negative regulation of metabolic process and molecular function (Fig. 5). There may be genetic programs present in CSD-R microglia that prevent the establishment of inflammatory phenotypes present in CSD-S microglia. Of the few transcripts unique to CSD-R microglia, several are implicated in regulating cellular and behavioral plasticity to emotional stimuli. For instance, increased expression of *Fosb* in brain reduces an animal’s sensitivity to the deleterious effects of chronic stress and promotes stress resilience^7^. Similar phenomenological anxiolytic effects have been documented with *Dnmt3a* overexpression^36^. *CDK5 regulatory subunit 1* (*Cdk5r1)*, the rate-limiting factor for CDK5 expression, has been linked to suppression of inflammatory proteins^77^. Likewise, *Dnmt3a* expression has been linked to the anti-inflammatory profile induced by IL-4 stimulated macrophages^78^, and was recently shown to restrain degranulation and cytokine production in stimulated mast cells^79^. Links to inflammatory suppression in microglia have not been explored, but these findings suggest viable avenues for further research. The presence of numerous resiliency genes may allow these cells to cope with DAMPs, perhaps by stabilizing gene expression during inflammatory events, thereby increasing their threshold for activation.

In summary, susceptibility to chronic psychosocial stress in this study closely correlated with particular microglial activation states revealed by microarray analysis and authenticated by histochemical assays. The aggregate changes in CSD-S microglia were similar to those resulting from LPS administration, neurodegenerative disease states, albumin infusion, and other CNS-centric inflammatory challenges. The similarities between peripheral and central triggers of the CNS immune response underscore the need to better understand immune-brain communication and the role of the BBB. Our findings suggest a novel mechanism of neuroinflammation in chronically stressed animals, perhaps precipitated by leakage of blood-borne substances into brain parenchyma, that occurs only in animals that show susceptibility to CSD. The transcriptional profile of susceptible microglia was highly enriched for pathways that indicative of CNS healing to sterile injury including inflammation, debris clearance, and wound resolution. The CSD-induced leakage of intravascular substances into brain parenchyma demonstrates a novel mechanism of neuroinflammation in chronically stressed animals. We still don’t know why animals fall into one or another category of stress susceptibility or resilience, but we do know that these outcomes have unique immunological signatures. Future work will test the causality of these mechanisms in precipitating the depressive effects of stress.

## Methods

### Animals

All procedures were approved by the NIMH Institutional Animal Care and Use Committee and conducted in accordance with NIH guidelines. Experiments were performed using male C57BL/6N (NIH/NCI/Division of Cancer Treatment, Frederick, MD), *Cx3cr*^*gfp/wt*^ (Jackson Laboratories, model B6.129P-*Cx3cr1*^*tm1Litt*^/J backcrossed onto a C57BL/6N background and bred in-house), and *Ubc*^*gfp/gfp*^ mice (Jackson, model C57BL/6-Tg(UBC-GFP)30Scha/J, bred in-house). CD-1 (Charles River Laboratories) mice used as aggressors were retired 4–6 month-old male breeders. All animals were housed in 12-h light/dark cycle with lights off at 9:00 AM. Food and water were provided *ad libitum*. Testing was done in the dark phase in mice aged 12–14 weeks.

### Chronic Social Defeat (CSD)

As in our previous studies^17,80,81^, an experimental intruder C57BL/6 or mutant mouse was placed into a resident CD-1 mouse’s home cage (polycarbonate cage 24.0 × 46.0 × 15.5 cm; Lab Products) into which a 5.5-mm-thick perforated transparent polycarbonate partition had been placed down the middle to separate the pair. Because wounding can precipitate numerous confounds, including opportunistic bacterial responses, peripheral immune activation, and splenomegaly^82^, mandibular incisors of the CD-1 mouse were trimmed with blunt scissors to prevent wounding injuries to the subordinate mouse. Defeats commenced after a 2-day accommodation period. The partition was removed for 5 min/day at approximately 11:00 AM to allow agonistic encounters between the mice. Defeat sessions were monitored to ensure that defeats reliably occurred^81^. The 24 h/day dyadic social housing exposed the defeated mouse to continuous psychological stress via sensory interaction with the aggressor. After 14 days of CSD, mice were killed 2 h after the last defeat and tissues harvested. Home-caged (HC) control mice were pair-housed in a divided 14.0 × 35.5 × 13.0 cm polycarbonate cage (Tecniplast) with one mouse on each side of the perforated clear divider, which remained in place throughout.

### Behavioral analysis

For most experiments, mice were phenotyped four days prior to harvest. CSD and HC mice were tested for affective changes using two tests that assess anti-social and anhedonic behaviors. 4 h separated each test. Automated tracking and scoring of behavior (TopScan; Cleversystems) was done^83^.

#### Social interaction (SI)

Mice were placed in a 50 × 50 cm open-field arena containing two perforated Plexiglas cylinders (10-cm diameter). One cylinder contained an unfamiliar aggressor CD-1 mouse used during CSD, and the other was empty. Test mice were placed in the open field and allowed to explore for 30 min. SI quotients were defined as a ratio of duration investigating the cylinder with versus without the CD-1 mouse.

#### Urine scent marking (USM)

As described previously^83^, a spot of urine from an estrous female mouse was placed in one corner of blotting paper covering the open-field floor. Test mice were placed in the arena and allowed to freely explore and scent mark for 10 min. The urine tracks were revealed by ninhydrin staining, the sheets photographed and digitally binarized, and the marked areas quantified using ImageJ^84^ (http://rsb.info.nih.gov/ij/). Dividing the area of marks in a circle within 10 cm of the female urine spot by the total area of marks in the arena provided the marking preference score.

#### Phenotype Stratification

SI and USM were used to stratify stressed populations into susceptible (CSD-S) and resilient (CSD-R) subgroups. Despite an overall reduction in social interaction, socially defeated mice showed a large variation in SI scores; most HC mice prefer interacting with a social target than an empty enclosure. Following exposure to stress, mice were characterized as susceptible (defined as a SI < 1.2) or resilient (defined as a SI ≥ 1.2), based on previously published and pharmacologically validated data^83^. Stressed subgroups determined by SI were further validated using USM. A resilient mouse as determined by SI ≥ 1.2 must show USM preference greater than 20%. Mice not corresponding to either criterion were excluded from the experiment.

### Microglia isolation

Described previously^17^, whole brains minus cerebellum were dissected from phenotyped HC and CSD mice perfused with 0.9% saline. Single-cell suspensions were created through enzymatic digestion using the Neural Tissue Dissociation Kit (Miltenyi Biotec) for 35 min at 37 °C. Cells suspended in 70% Percoll layered under 30% Percoll were centrifuged at 1000 × g at 10 °C for 40 min. The supernatant containing myelin was removed, and cells were collected at the 30/70% Percoll interface. Cells were magnetically labeled with CD11b microbeads (Miltenyi Biotec). Using the manufacturer’s guidelines, CD11b^hi^ cells were separated in a magnetic field using MS columns (Miltenyi Biotec). Each brain extraction yielded approximately 3 × 10^5^ viable cells.

### Microarray

Brain CD11b^hi^ cells isolated with MACS were placed in 500 µl TRIzol (Invitrogen), homogenized with a 25-gauge needle and syringe, and then stored at −80 °C. Samples (*n* = 6 HC, *n* = 6 CSD-S, *n* = 9 CSD-R) were prepared according to Affymetrix protocols (Affymetrix, Inc.) and as described previously^64^. RNA quality and quantity was ensured using the Bioanalyzer (Agilent, Inc.) and NanoDrop (Thermo), respectively. 200 ng of total RNA was prepared according to Affymetrix protocols and hybridized to Affymetrix MoGene 2.0 ST chips. The chips were washed and stained by the Affymetrix Fluidics Station using the standard format and protocols. Probe-level data for 29,215 mouse gene fragments per hybridized cDNA were generated using an Affymetrix Gene Chip Scanner 3000. The resulting.cel files were imported into Partek Genomics Suite version 6.6 (Partek, St Louis, MO, USA) and RMA (Robust Multichips Analysis; part of Partek Genomics Suite software, version 6.6 beta) background correction and quantile normalization was performed. A Median Polish was done for probeset summarization, and the data were Log2 transformed. Data quality was assessed by visual inspection with a Tukey box plot, covariance-based Principal Component Analysis (PCA), scatter plot, and correlation-based Heat Map.

### Bioinformatics Analysis

Normalized data sets were compared in Partek by ANOVA with false discovery rate (FDR) correction (*q* < 0.05) to assess differential transcript expression across behavioral phenotypes. Data were visualized by heatmap with transcripts and samples organized by hierarchical clustering on log transformed and Z-score transformed data. Both samples and genes were hierarchically clustered based on the Euclidean and Average Linkage method. Heatmaps (samples on rows and genes on columns) were drawn from the different clusters identified. The colors are based on relative standardized expression values (mean of 0, standard deviation of 1; performed on the fly), with blue indicating low and red indicating high levels of a variable (i.e. low or high expression levels). This analysis was performed within Partek Genomics Suite version 6.6. Unsupervised cluster analysis was then performed on differentially expressed transcripts to examine gene coexpression relationships across all phenotypes. A pairwise transcript-to-transcript matrix was first calculated in Miru (Kajeka Ltd, Edinburgh, UK).) using a Pearson correlation threshold (*r* = 0.8). The resulting matrix was then visualized as a network graph where nodes represent individual genes and edges represent correlated expression patterns. The network graph was clustered into discrete groups of transcripts using Markov Clustering with inflation set to 2.2 and 10 nodes as the minimum cluster size. The composition and biological functions of four major clusters were then interpreted using enrichment analysis for Gene Ontology (GO) terms in DAVID^85^ (https://david.ncifcrf.gov). Gene lists were uploaded into DAVID and using the GOTERM_BP_FAT annotation category, enriched GO terms were collected for each of the four major clusters. Default settings were used for analysis with enrichment based on *p* < 0.05 with Benjamini correction. Enriched GO terms were imported into REViGO^86^ (http://revigo.irb.hr/), redundant terms removed, and clustered according to semantic similarity measures then visualized in semantic space, revealing representative GO terms.

We mined published microarray data sets to further define phenotypes in the present data using Gene Set Enrichment Analysis (GSEA)^87^ (http://broadinstitute.org/GSEA) according to methods described previously^88^. The GSEA algorithm computes a ranked list of all genes from a microarray comparison between two conditions and identifies whether individual members of an *a priori* functionally defined gene set (black vertical bars) are enriched at either the top (red area) or bottom of the ranked genes (blue area) or randomly distributed across the whole ranked gene list, using a modified Kolmogorov-Smirnov statistic and generating an enrichment score. The enrichment score represents the degree to which a specific set is represented at the extremes (high or low) of the entire list. FDR *q*-value was set at <0.05 and statistical significance adjusted for multiple hypothesis testing was *p* < 0.05. A gene set-based permutation test of 1000 permutations was applied and genes were ranked according to Student’s *t* statistic. All other parameters were set to GSEA defaults.

Microarray data sets from microglia stimulated with either LPS or IL-4 were mined to determine immunophenotypes of cells in the present data set^46^. Raw expression data from NCBI GEO Data sets (GSSE49329) were imported into Partek and normalized with the microglia data. Non-overlapping genes significantly upregulated by LPS (>5-fold, FDR *q* < 0.05) or IL4 (>1.5-fold, FDR *q* < 0.05) were determined. These gene lists were then overlaid on our data set using GSEA. The expression profile for the top 20 most significantly enriched, non-overlapping transcripts for each stimulus was visualized by heat map using log-transformed and *z* score−transformed data.

Ingenuity Pathways Analysis (IPA; Qiagen, Inc.) was used for revealing top upstream regulators for each Markov cluster generated by Miru, and to further examine highly interconnected nodes within major clusters as putative hub genes. Of several tools available for mining array data, IPA uses a high percentage of curation by experts for multiple sources of information (see https://www.qiagenbioinformatics.com/products/ingenuity-pathway-analysis/).

### Quantitative PCR

Quantitative RT-PCR was performed on a subset of the samples (*n* = 6 HC, *n* = 5 CSD-S, *n* = 5 CSD-R) to validate select candidate genes from our microarray data. Total RNA was extracted using a commercial kit (Qiagen), reverse transcribed using a Superscript III First Strand cDNA Synthesis Kit (Invitrogen) and real-time RT-PCR with 2× SYBR Green Master Mix (Bio-Rad) was performed using the Bio-Rad iCycler. All primers were validated and sequenced prior to this experiment including lipocalin 2 [(*Lcn2*, NM008491) F: *CTGTCCCCTGAACTGAAGGA*, R: *AGGAAAGATGGAGTGGCAGA*]; matrix metalloprotease 8 [(*Mmp8*, NM008611) F: *TTTGATGGACCCAATGGAAT*, R: *GAGCAGCCACGAGAAATAGG*]; and matrix metalloprotease 9 [(*Mmp9*, NM013599.4) F: *TTCCAGTACCAAGGCCTCTC*, R: *TCACACGCCAGAAGAATTTG*]. TATA-binding protein (*Tbp*, NM013684.3) was used to normalize quantification of the mRNA target (F: *GACCCACCAGCAGTTCAGTA*, R: *AAACACGTGGATAGGGAAGG*). Relative expression levels were determined by the 2^−ΔΔCt^ method, and the data were expressed as the fold change relative to the HC sample.

### Microglial culture and immunocytochemistry

For array verification and phagocytosis assay, purified microglial cells were suspended in DMEM F12 + GlutaMax supplemented with 10% qualified fetal bovine serum and plated at 5 × 10^4^ cells/well on Lab-Tek Chamber slides (Nalgene Nunc) at 37 °C and 5% CO_2_. Culture duration was 24 h for MACS validation and 3 h for phagocytosis assay. At the end of the assay, cells were washed and fixed with 4% paraformaldehyde (PFA) for 20 min. Cells were blocked in 10% goat serum in 0.1 M phosphate-buffered saline (PBS) containing 0.3% Triton X-100 and 0.5% BSA for 1 h before adding primary antibodies prepared in 0.1% Triton X-100 (Sigma). For array verification, cells were stained with microglial markers rabbit anti-Iba1 (Cat. #019-19741, RRID: AB_839504, Wako Chemicals, 1:1000) and rat anti-F4/80 (Cat. #565410, RRID:AB_394533, BD Pharmingen, 1:500). For the phagocytosis assay, cells were stained with the microglia marker rabbit anti-CX3CR1 (Cat #TP501, RRID AB_10892355, Torrey Pines Biolabs, 1:1000). After 1-h incubations, cells were washed for 10 min three times at RT in 0.1% Triton X-100 (Sigma) in PBS, followed by incubation in secondary antisera directed against the host species (Life Technologies, 1: 1,000) for 1 h at RT: anti-rat Alexa-Fluor 488 (Cat. #A11006), anti-rabbit Alexa-Fluor 555 (Cat. #A21428) for array verification or anti-rabbit Alexa-Fluor 488 (Cat. #A11034) for the phagocytosis assay. Cells were washed again with 0.1% Triton X-100 in 0.1 M PBS (10 min, three times), counterstained with DAPI for 1 min, washed with PBS, and coverslipped with Fluoromount (Sigma). Cells were analyzed on a Zeiss 780 confocal microscope with the following parameters for array verification. 488 nm argon laser at 10% transmission was used for Alexa488, F4/80; DPSS 561 nm laser at 8% transmission was used for Alexa555, Iba1; and a 405 nm Diode laser at 8% transmission was used for DAPI. Z-stacked images (0.7 µm/section) were captured with a Zeiss EC Plan-Neofluar 40x (1.3 aperture) oil immersion objective.

### Phagocytosis assay

Described previously^17^, 2 h after the last defeat, mice were perfused with saline, and microglia isolated with CD11b microbeads were incubated for 3 h in culture conditions described above. During the 3 h incubation, neural cells from separate HC mice were isolated using enzymatic digestion and Percoll gradients and exposed to 254 nm ultraviolet radiation (UV) for 20 min. Apoptosis was verified with trypan blue. UV-exposed cells were then stained with 5 µl of 5(6)-carboxytetramethylrhodamine, succinimidyl ester (5(6)-TAMRA, SE) (Invitrogen) to mark phagocytosed debris^89^, and washed thoroughly with cold PBS before they were fed to microglia. After a further 3-h incubation at 37 °C, media was removed and cells were washed, fixed with 4% PFA, and stained for CX3CR1 using methods described above. 25 images from each condition were randomly captured from each condition with confocal microscopy. A phagocytic index was calculated by dividing the total area of phagocytosed TAMRA-labeled apoptotic cells by the total area of microglial (CX3CR1-positive) cells using NIH ImageJ software. The following confocal parameters were used for the phagocytosis assay: z-stacked images (0.7 µm/section) were captured with a Zeiss Plan-Apochromat 63x (1.4 aperture) oil immersion objective using a 488 nm argon laser (10% transmission) to image Alexa488, CX3CR1; DPSS 561 nm laser (12% transmission) to image TAMRA; and a 405 nm diode laser (10% transmission) to image DAPI.

### BBB changes: Fluorescein spectrophotometric assay

BBB permeability was further evaluated with sodium fluorescein (Cat #F6377-100G, Sigma Aldrich). Briefly, and described previously^90^, 2 h after the last defeat, 10 mg sodium fluorescein in 0.1 ml sterile saline was administered i.p. to mice and allowed to circulate for 45 min. Mice were anesthetized, blood drawn from heart and placed into serum separator tubes (Sarstedt), then perfused with 50 ml saline. Brain was removed, weighed, and homogenized in 1 ml sterile PBS. Protein was precipitated from brain and serum samples to remove potential background fluorescence during a 24-h incubation with 20% trichloroacetic acid (TCA) at 4 °C. Samples were centrifuged at 10,000 × *g* for 15 min, and supernatant was diluted 1:1 with borate buffer (50 mM, pH 10). Samples, run in triplicate with a standard curve, were analyzed on a fluorimeter (Victor2, Perkin Elmer) (Ex: Em, 480 nm: 538 nm). The amount of BBB permeability was measured as the ratio (w/v) of sodium fluorescein in a gram of brain tissue per the amount in a milliliter of serum.

### BBB changes: Alexa Fluor 488 Dextran histochemistry

Fixable Alexa Fluor 488-dextran was used for the histochemical evaluation of BBB permeability. 2 h after the last defeat and 30 min prior to harvest, isoflurane-anesthetized mice were intravenously injected with 0.1 ml of 1 mg/ml solution of fixable Alexa Fluor 488-dextran (cat. No. D22910, MW = 10 kDa; Thermo Fisher Scientific). Two min prior to harvest, mice were injected i.v. with 0.1 ml DyLight 594-labeled Tomato Lectin (Vector Labs). Deeply anesthetized mice were perfused with saline followed by ice-cold 4% PFA. Brains were removed, post-fixed overnight followed by 25% sucrose PBS for 24 h. Coronal brain slices (30-μm thick) were collected on a freezing microtome. Free-floating sections were washed, slide mounted, and counterstained with DAPI. Using confocal (Zeiss 780) microscopy, extravasation of the Alexa Fluor 488-dextran was defined as presence of specific fluorescence occurring outside the vessel lumen. Ten sections per animal were examined by a blind observer. The following confocal parameters were used to capture Alexa Fluor 488-dextran histochemistry: images were acquired with a Zeiss 20x Plan Apochromat Objective (0.8 aperture) using a 488 nm argon laser (10% transmission) to image Alexa488, dextran; DPSS 561 nm laser (8% transmission) to image alexa594, tomato lectin; and a 405 nm diode laser (8% transmission) to image DAPI.

### Production of reactive oxygen species (ROS)

Oxidative stress in the form of ROS production was measured using dihydroethidium (DHE), the reduced form of ethidium bromide. DHE freely permeates cell membranes and is highly reactive, making it ideal to detect cytosolic superoxide^91^. Upon reaction with superoxide anions, it forms a red fluorescent product (2OH-ethidium) that intercalates with DNA^92^. DHE (Cat #D11347, Molecular Probes, Life Technologies) dissolved 1 mg/ml in 100% DMSO, then diluted 1:1 with sterile saline, was administered at 2.5 µg/kg i.p. to *Cx3cr1*^*gfp/wt*^ mice. 3 h after administration, mice were perfused with saline followed by ice-cold 4% PFA. Brains were removed and frozen, sectioned at 30 µm with a freezing microtome, and counterstained with DAPI. Slide-mounted sections were coverslipped after drying with PVA-DABCO. Areas of interest were captured with a confocal microscope, 2OH-Ethidium (Ex:Em 405:600–650 nm) and DAPI (Ex:Em 405: 450–500 nm) were imaged with 405 nm diode laser at 10% transmission; GFP was imaged with a 488 nm argon laser at 15% transmission. Z-stacked images (0.7 µm/section) were captured with a Zeiss EC Plan-Neofluar 40x (1.3 aperture) oil immersion objective and analyzed for DHE-positive cells and DHE colocalization in GFP-positive microglia by a blind observer using NIH ImageJ.

### Extravasation tests: Flow cytometry

Single brain cell suspensions were created through enzymatic digestion (Neural Tissue Dissociation Kit; Miltenyi Biotec) and Percoll gradients as described above. Cells isolated from wildtype mice were incubated on ice for 30 min with anti-mouse CD16/CD32 (Cat. #553141, Clone 2.4G2, BD Pharmingen) to block Fc receptors, and then incubated on ice for 30 min with a mix of fluorochrome-conjugated anti-mouse antibodies CD11b-APC (Cat. #130-091-828, Miltenyi Biotec) and CD45-PE (Cat. #103106, Biolegend).

In adoptive transfer experiments, spleens were also enumerated for GFP-positive cells to determine successful colonization. Spleens were removed prior to perfusion, and single cell suspensions were created using a gentleMACS dissociator (Miltenyi Biotec). Cell suspensions were passed through a 40-μm cell strainer, concentrated using a 30/70% discontinuous Percoll gradient as described above, and examined for GFP fluorescence using flow cytometry (MoFlo Astrios; Beckman Coulter). Data were analyzed with FlowJo software (TreeStar).

### Extravasation tests: Adoptive transfer of GFP-positive cells and positive inflammation control experiment

As described previously^17^, spleen cells from *Ubc*^*gfp/gfp*^ mice with ubiquitous expression of GFP were adoptively transferred into wildtype mice to determine if peripheral monocytes are recruited into brain after stress. Splenocytes from normal *Ubc*^*gfp/gfp*^ donor mice were isolated and purified as described above, then resuspended in physiological PBS and injected retro-orbitally at a concentration of 25 million cells per host in a volume of 0.15 ml. Host CSD mice were exposed to defeat for 10 days, phenotyped, given cell transfer, exposed to a further four days of CSD, and killed for tissue harvest. HC mice received transfer four days prior to endpoint analysis.

A positive control for demonstrating splenocyte infiltration into brain was generated as described previously^17^. Unstressed mice received *Ubc*^*gfp/gfp*^ cell transfer followed 24 h later with 0.1 mg/kg lipopolysaccharide (LPS) (Cat. #L2880, serotype 055:B5, Sigma) in sterile saline administered i.p. 24 h later, 100 ng of IL-1β (R&D, cat. #401-ML) was administered intracisternally into the cerebrospinal fluid. 24 h later, spleen and brain were examined for presence of GFP-positive cells by flow cytometry.

### Experimental Design and Statistical Analysis

Data were summarized as mean ± SEM, and differences among experimental conditions were considered statistically significant when the *p* value was ≤0.05. One-way ANOVAs, analyzed using SPSS software (version 23) were applied to assess between-subject comparisons along the variables behavioral condition versus e.g., behavioral or biological assay score. Post-hoc tests were done with Bonferroni’s procedure. Statistical analysis for qPCR and microarray is described above.

### Data availability

The sequencing data have been deposited at https://www.ncbi.nlm.nih.gov/geo/query/acc.cgi?acc=GSE107941 under accession number GSE107941. Other data are shown in Supplemental Tables 1–4.

## Electronic supplementary material

Supplementary information

Table 1

Table 2

Table 3

Table 4
