# Supplementary material for: Decoding microglia responses to psychosocial stress reveals blood-brain barrier breakdown that may drive stress susceptibility
Source: Sci Rep. 2018 Jul 26;8:11240. doi: 10.1038/s41598-018-28737-8 (PMC6062609; doi:10.1038/s41598-018-28737-8)
Supplement: Supplementary file 1 — Supplementary information [file 41598_2018_28737_MOESM1_ESM.docx]

**Supplementary Information**

Decoding microglia responses to psychosocial stress reveals blood-brain barrier breakdown that may drive stress susceptibility

Authors: Michael L. Lehmann*^1^, Thaddeus K. Weigel^1^, Hannah A. Cooper^1^, Abdel G. Elkahloun^2^, Stacey L. Kigar^1^, and Miles Herkenham^1^

1. Section on Functional Neuroanatomy, Intramural Research Program, National Institute of Mental Health, NIH, Bethesda, MD 20892 USA

2. Division of Intramural Research Programs Microarray Core Facility, National Institutes of Health, Bethesda, MD, 20892 USA

*Corresponding author at: Bldg. 35, Rm. 1C911, Bethesda, MD 20892-3724. *E-mail address:* [michael.lehmann@nih.gov](mailto:michael.lehmann@nih.gov)

**Table Descriptions**

**Supplemental Table 1:** Full annotation of gene transcripts found in clusters 1-4.

**Supplemental Table 2:** Full annotation of Gene Ontology functions associated with clusters 1-4.

**Supplemental Table 3:** Full annotation of Gene Ontology enrichment is shown for clusters 1-4.

**Supplemental Table 4:** Full annotation of node connection in Cluster 1 and 2. The number of connection a transcript has with other nodes in a cluster is called “Hubness.” Highly interconnected nodes are called hub genes. Pathway Analysis in Ingenuity was used to identify upstream regulators and are fully annotated in separate tabs for cluster 1 and 2.

|  |
| --- |
|  |
|  |
